# The Preclinical Natural History of Serous Ovarian Cancer: Defining the Target for Early Detection

**DOI:** 10.1371/journal.pmed.1000114

**Published:** 2009-07-28

**Authors:** Patrick O. Brown, Chana Palmer

**Affiliations:** 1 Department of Biochemistry, Stanford University School of Medicine, Stanford, California, United States of America; 2 Howard Hughes Medical Institute, Stanford, California, United States of America; 3 Canary Foundation, San Jose, California, United States of America; University of Cambridge, United Kingdom

## Abstract

**Background:**

Ovarian cancer kills approximately 15,000 women in the United States every year, and more than 140,000 women worldwide. Most deaths from ovarian cancer are caused by tumors of the serous histological type, which are rarely diagnosed before the cancer has spread. Rational design of a potentially life-saving early detection and intervention strategy requires understanding the lesions we must detect in order to prevent lethal progression. Little is known about the natural history of lethal serous ovarian cancers before they become clinically apparent. We can learn about this occult period by studying the unsuspected serous cancers that are discovered in a small fraction of apparently healthy women who undergo prophylactic bilateral salpingo-oophorectomy (PBSO).

**Methods and Findings:**

We developed models for the growth, progression, and detection of occult serous cancers on the basis of a comprehensive analysis of published data on serous cancers discovered by PBSO in *BRCA1* mutation carriers. Our analysis yielded several critical insights into the early natural history of serous ovarian cancer. First, these cancers spend on average more than 4 y as in situ, stage I, or stage II cancers and approximately 1 y as stage III or IV cancers before they become clinically apparent. Second, for most of the occult period, serous cancers are less than 1 cm in diameter, and not visible on gross examination of the ovaries and Fallopian tubes. Third, the median diameter of a serous ovarian cancer when it progresses to an advanced stage (stage III or IV) is about 3 cm. Fourth, to achieve 50% sensitivity in detecting tumors before they advance to stage III, an annual screen would need to detect tumors of 1.3 cm in diameter; 80% detection sensitivity would require detecting tumors less than 0.4 cm in diameter. Fifth, to achieve a 50% reduction in serous ovarian cancer mortality with an annual screen, a test would need to detect tumors of 0.5 cm in diameter.

**Conclusions:**

Our analysis has formalized essential conditions for successful early detection of serous ovarian cancer. Although the window of opportunity for early detection of these cancers lasts for several years, developing a test sufficiently sensitive and specific to take advantage of that opportunity will be a challenge. We estimated that the tumors we would need to detect to achieve even 50% sensitivity are more than 200 times smaller than the clinically apparent serous cancers typically used to evaluate performance of candidate biomarkers; none of the biomarker assays reported to date comes close to the required level of performance. Overcoming the signal-to-noise problem inherent in detection of tiny tumors will likely require discovery of truly cancer-specific biomarkers or development of novel approaches beyond traditional blood protein biomarkers. While this study was limited to ovarian cancers of serous histological type and to those arising in *BRCA1* mutation carriers specifically, we believe that the results are relevant to other hereditary serous cancers and to sporadic ovarian cancers. A similar approach could be applied to other cancers to aid in defining their early natural history and to guide rational design of an early detection strategy.

*Please see later in the article for Editors' Summary*

## Abstract 2

Pat Brown and colleagues carry out a modeling study and define what properties a biomarker-based screening test would require in order to be clinically useful.

## Editors' Summary

**Background:**

Every year about 190,000 women develop ovarian cancer and more than 140,000 die from the disease. Ovarian cancer occurs when a cell on the surface of the ovaries (two small organs in the pelvis that produce eggs) or in the Fallopian tubes (which connect the ovaries to the womb) acquires genetic changes (mutations) that allow it to grow uncontrollably and to spread around the body (metastasize). For women whose cancer is diagnosed when it is confined to the site of origin—ovary or Fallopian tube—(stage I disease), the outlook is good; 70%–80% of these women survive for at least 5 y. However, very few ovarian cancers are diagnosed this early. Usually, by the time the cancer causes symptoms (often only vague abdominal pain and mild digestive disturbances), it has spread into the pelvis (stage II disease), into the space around the gut, stomach, and liver (stage III disease), or to distant organs (stage IV disease). Patients with advanced-stage ovarian cancer are treated with surgery and chemotherapy but, despite recent treatment improvements, only 15% of women diagnosed with stage IV disease survive for 5 y.

**Why Was This Study Done?:**

Most deaths from ovarian cancer are caused by serous ovarian cancer, a tumor subtype that is rarely diagnosed before it has spread. Early detection of serous ovarian cancer would save the lives of many women but no one knows what these cancers look like before they spread or how long they grow before they become clinically apparent. Learning about this occult (hidden) period of ovarian cancer development by observing tumors from their birth to late-stage disease is not feasible. However, some aspects of the early natural history of ovarian cancer can be studied by using data collected from healthy women who have had their ovaries and Fallopian tubes removed (prophylactic bilateral salpingo-oophorectomy [PBSO]) because they have inherited a mutated version of the *BRCA1* gene that increases their ovarian cancer risk. In a few of these women, unsuspected ovarian cancer is discovered during PBSO. In this study, the researchers identify and analyze the available reports on occult serous ovarian cancer found this way and then develop mathematical models describing the early natural history of ovarian cancer.

**What Did the Researchers Do and Find?:**

The researchers first estimated the time period during which the detection of occult tumors might save lives using the data from these reports. Serous ovarian cancers, they estimated, spend more than 4 y as in situ (a very early stage of cancer development), stage I, or stage II cancers and about 1 y as stage III and IV cancers before they become clinically apparent. Next, the researchers used the data to develop mathematical models for the growth, progression, and diagnosis of serous ovarian cancer (the accuracy of which depends on the assumptions used to build the models and on the quality of the data fed into them). These models indicated that, for most of the occult period, serous cancers had a diameter of less than 1 cm (too small to be detected during surgery or by gross examination of the ovaries or Fallopian tubes) and that more than half of serous cancers had advanced to stage III/IV by the time they measured 3 cm across. Furthermore, to enable the detection of half of serous ovarian cancers before they reached stage III, an annual screening test would need to detect cancers with a diameter of 1.3 cm and to halve deaths from serous ovarian cancer, an annual screening test would need to detect 0.5-cm diameter tumors.

**What Do These Findings Mean?:**

These findings suggest that the time period over which the early detection of serous ovarian cancer would save lives is surprisingly long. More soberingly, the authors find that a test that is sensitive and specific enough to take advantage of this “window of opportunity” would need to detect tumors hundreds of times smaller than clinically apparent serous cancers. So far no ovarian cancer-specific protein or other biomarker has been identified that could be used to develop a test that comes anywhere near this level of performance. Identification of truly ovarian cancer-specific biomarkers or novel strategies will be needed in order to take advantage of the window of opportunity. The stages prior to clinical presentation of other lethal cancers are still very poorly understood. Similar studies of the early natural history of these cancers could help guide the development of rational early detection strategies.

**Additional Information:**

Please access these Web sites via the online version of this summary at http://dx.doi.org/10.1371/journal.pmed.1000114.

- The US National Cancer Institute provides a brief description of what cancer is and how it develops and information on all aspects of ovarian cancer for patients and professionals. It also provides a fact sheet on BRCA1 mutations and cancer risk (in English and Spanish)
- The UK charity Cancerbackup also provides information about all aspects of ovarian cancer
- MedlinePlus provides a list of links to additional information about ovarian cancer (in English and Spanish)
- The Canary Foundation is a nonprofit organization dedicated to development of effective strategies for early detection of cancers including ovarian cancer.

## Introduction

In 2007, ovarian cancer is estimated to have killed more than 15,000 women in the United States [1] and more than 140,000 worldwide [2]. The majority of these deaths were from ovarian cancer of the serous histological type. The other histological subtypes of ovarian cancer differ substantially in their molecular features and natural history and can be considered distinct diseases [3],[4]. Ovarian carcinomas of the serous histological type are an attractive target for early detection as they are rarely detected before they reach an advanced stage, when they are highly lethal [5]. The success of early intervention strategies for other cancers, including prostate, colon, and cervical cancer, gives us hope that early detection of ovarian cancer could be an effective way to save lives [6].

In order to save lives by early detection we need to detect and eliminate tumors that would otherwise ultimately be lethal. We know surprisingly little about the target for early detection of serous ovarian cancer. What do lethal serous ovarian cancers look like during the “window of opportunity”—the period before they become incurable? It is sometimes implicitly assumed that the ovarian cancers that present clinically at early stages represent the precursors to cancers that present at advanced stages, but the evidence argues strongly against this model. For example, few ovarian cancers that present at stage I are of serous histology, yet most ovarian cancers that present at stage III or IV are serous [4],[5]. Clearly it would be a mistake to assume that nonserous stage I cancers transform into advanced-stage serous cancers. Similarly, we cannot simply assume that advanced serous cancers, although histologically similar, were once equivalent to the rare serous cancers that are clinically diagnosed at stage I. By defining the what, when, and where of preclinical ovarian cancer, we can begin to rationally design an effective early detection strategy.

The ideal approach to defining the early natural history of a cancer would be to carefully observe tumor development from genesis through late stage without intervening. While this ideal study is not feasible, we can still clarify many aspects of the early natural history of ovarian cancer by taking advantage of data from prophylactic bilateral salpingo-oophorectomies (PBSOs) (surgical removal of both fallopian tubes and ovaries). Many apparently healthy women with a family history of ovarian cancer choose to undergo this procedure, which has been proven to reduce mortality from ovarian cancer [7]–[10]. In a small fraction of these women, an unsuspected cancer is discovered [7]–[26]. We can estimate the duration of the window of opportunity for early detection by comparing the prevalence of nonadvanced occult serous cancers in these PBSO specimens with the incidence of serous ovarian cancer in a comparable population [27]. Furthermore, the size and stage distribution of these occult tumors provides a basis for modeling the growth and progression of tumors that an early detection test must identify.

Here, we present a summary and analysis of available reports describing the frequency and characteristics of occult serous cancers discovered in prospective series of PBSOs in women at known genetic risk of serous ovarian cancer. We focused our analyses on women with mutations in the *BRCA1* gene, the most common genetic risk factor, for the sake of homogeneity and the ability to synthesize and compare data from multiple reports [8],[28],[29]. Using these data, we developed a model for the preclinical natural history of serous ovarian cancer and considered its implications for early detection. Our findings help to define the performance requirements for early detection biomarkers and screening modalities.

## Methods

**Figure 1:**
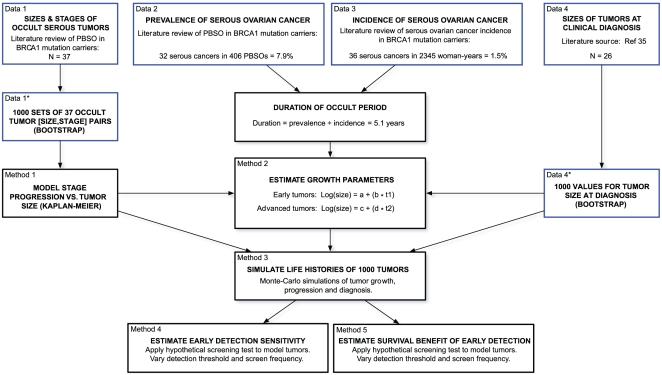
Schematic of our modeling strategy. The boxes outlined in blue represent the data that were used to build the model. The boxes outlined in black represent critical steps in the modeling procedure. Data sources and modeling methods are described in Methods.

Our modeling strategy is schematized in Figure 1. The data sources and details of the methods are described below. Further details on the modeling methods and the full R [30] code are available in Texts S1, S2, S3, S4, S5.

### Data Sources

#### Sizes and stages of occult tumors

Sizes and stages of occult tumors (Figure 1, Data 1) were taken directly from the PBSO studies when possible. Twelve tumors that were described as microscopic (detected by microscopic examination but not by gross examination), but for which specific dimensions were not provided, were assumed to have the same size distribution as the 21 tumors in these studies that were also described as microscopic (by the same definition) for which the specific dimensions were reported. In four cases where multiple tumors were found in a single patient, we assumed that the tumors were spheres of the specified diameter(s) and used as our “diameter” estimate the diameter of a sphere with a volume equivalent to the total estimated volume of the tumors. Complete size and stage data as reported in the referenced studies are reported in Table S1.

#### Prevalence estimates

Our prevalence estimates were based on a comprehensive review of all available data from published series of PBSOs (Figure 1, Data 2) [8]–[18],[20]–[22],[24],[31],[32]. Our criteria for study inclusion were: (1) the prophylactic procedure included removal and microscopic examination of both ovaries and both Fallopian tubes; (2) tumor histology was reported; (3) patient genotypes were reported; (4) genotyping was done prior to cancer diagnosis. The requirement for careful examination of both Fallopian tube and ovaries was based on evidence that serous “ovarian” cancers can arise initially in either the Fallopian tubes or the ovaries [20],[31],[33],[34]. Occult cancers were defined as cancerous lesions that were neither detected nor suspected prior to surgery, including carcinoma in situ (CIS) and stage I–IV cancers, but not atypical hyperplasia. Further details of the studies we reviewed for prevalence estimates are available in Table S1.

#### Incidence estimates

Our estimate of the incidence of serous ovarian cancer in *BRCA1* women was based on a comprehensive review of all available data from systematic studies of the incidence of ovarian cancer in high-risk populations (Figure 1, Data 3) [8]–[18],[20]–[22],[24],[31],[32]. Our inclusion criteria were: (1) the study design must be prospective; (2) patients must have been genotyped prior to cancer diagnosis. The mean age of the population on which the incidence calculation was based had demographics very similar to the set of patients who underwent PBSO (mean age 45.7 y for prevalence; 46.9 y for incidence). Further details of the studies we reviewed are available in Table S2.

#### Sizes of tumors at clinical diagnosis

We modeled the distribution of sizes at which tumors would be diagnosed in high-risk populations based on the size distribution of serous tumors detected in a large trial of screening for early detection (Figures 1, Data 4 and S1) [35]. Our rationale was that the high-risk women on whom our prevalence and incidence estimates were based were likely to have been more closely monitored than women in the general population. This study included sizes for 17 screen-detected tumors but failed to report sizes for nine tumors that were detected clinically between screens. These nine interval cancers were assumed to have diameters log-normally distributed with a mean of 8 cm; note that our sensitivity analyses indicated that variation in this value over a range from 4–12 cm had a negligible effect on results. For our estimates of early-detection screen performance in a normal-risk population, we assumed that in these women serous ovarian cancers present clinically at a diameter log-normally distributed with a mean of 8 cm (P. Shaw and B. Rosen, unpublished data; Figure S1).

### Confidence Intervals for Estimates of Serous Cancer Prevalence, Incidence, and Duration of Occult Intervals

#### Prevalence

We estimated the probability distribution of the underlying prevalence of serous cancer in women with *BRCA1* mutations, given the observed 32 cancers in 406 patients, using the binomial distribution (Figure S2A). The 2.5th percentile and 97.5th percentile of this distribution gave the bounds of the 95% confidence interval (CI) for the true prevalence.

#### Incidence

We estimated the probability distribution of the underlying incidence of serous cancer in women with *BRCA1* mutations, given the observed 36 serous cancers in 2,345 woman-years, using the binomial distribution (Figure S2B). The 2.5th percentile and 97.5th percentile of this distribution gave the bounds of the 95% CI for the true incidence.

#### Duration of the occult period

The mean duration of the occult period was estimated by prevalence/incidence (see Table S1 for raw data). We used the ratio of 10,000 pairs of (prevalence, incidence) values from the distributions described above to estimate the probability distribution of the mean duration of the occult period (Figure S2C). We used the same approach to calculate the probability distributions for the fraction of the occult period spent at CIS, stage I or stage II (i.e., the “window of opportunity” for early detection) (Figure S2D). The R [30] code used to make these CI calculations is available in Text S1.

### Modeling Stage Progression as a Function of Tumor Size

We modeled stage progression as a function of tumor size using a Kaplan-Meier analysis analogous to a conventional Kaplan-Meier analysis of progression as a function of time after diagnosis, but replacing “time after diagnosis” with “tumor size,” censoring observations at the size of the tumor at diagnosis (i.e., when discovered by PBSO) (Figure 1, Method 1). We assumed that serous tumors grow monotonically and that the likelihood of having progressed from early to advanced stage increases with time. For the primary analysis, we defined “early stage” to include CIS, stage I and stage II cancers, and “advanced stage” to include stage III and IV cancers. We estimated CIs for the Kaplan-Meier survival curves by carrying out the Kaplan-Meier analysis on 1,000 simulated datasets generated by bootstrap resampling (with normal kernel smoothing), of the 37 pairs of occult tumor (size, stage) values obtained from the PBSO studies (Figure 1, Data 1, see above). The tumors that were at an advanced stage when discovered by PBSO had actually progressed to advanced stage at some unknown time prior to their discovery. We therefore assumed that detection of these advanced tumors by PBSO occurred stochastically at times after progression to advanced stage uniformly distributed between zero and the average time between progression and clinical diagnosis. We could thus infer the sizes of these advanced-stage tumors at progression on the basis of: (1) tumor size at time of discovery by PBSO; (2) our estimate of the duration of the advanced-occult period; (3) our estimate of the growth rate of advanced tumors (see “Modeling tumor growth and progression” below).

We carried out a second Kaplan-Meier analysis of progression from (CIS and stage I) to (stages II, III, or IV) as a function of tumor diameter (Figure S3). We estimated the sizes of stage II, III, and IV tumors at progression from CIS or stage I based on: (1) the sizes of these tumors when discovered by PBSO; (2) our estimates of the average times spent at stage II or stage III+ prior to clinical presentation; (3) our estimate of the growth rates of early or advanced tumors, respectively. We again assumed that detection of tumors by PBSO occurred stochastically at times after progression to the stage at diagnosis, uniformly distributed between zero and the average duration of the tumor stage at detection by PBSO. The R code used for these analyses is available in Text S2.

### Modeling Tumor Growth

We used a Monte Carlo method to fit an exponential model for tumor growth, with separate growth rate parameters for early (CIS, stage I and stage II) and advanced (stage III and IV) tumors (Figure 1, Method 2).

Early stage tumors were assumed to grow exponentially such that: log(size) = *a* + (*b* * *t1*) where *a* = intercept (size at which the tumor is detectable by histopathology); *b* = exponential growth rate constant and *t1* is the time since the tumor became detectable by histopathology. To estimate *a* and *b*, we tested 625 pairs of (slope, intercept) values (25 slope × 25 intercept). For each pair of values, we modeled the growth of 1,000 hypothetical tumors according to the specified parameters and compared the resulting distribution of tumors to the observed data from PBSO studies. Specifically, we chose the slope and intercept values that gave a distribution of early-stage occult tumor sizes that best fit the sizes of early-stage tumors discovered by PBSO, and a duration that fit our estimate of the average duration of the early-stage occult period.

Advanced tumors were assumed to grow exponentially such that: log(size) = *c* + (*d* * *t2*) where *c* = log(tumor diameter at progression to advanced stage) obtained from the Kaplan-Meier analysis; *d* = [log(mean size at diagnosis [from [35]])−log(mean size at progression [from Kaplan Meir analysis])]/duration of advanced occult period; *t2* = time since progression. The R code used for this analysis is available in Text S2. Observed versus model-predicted sizes of occult serous ovarian cancers are shown in Figure S4.

### Simulating Model Tumor Life Histories

We used a Monte Carlo method to generate models for the life histories of 1,000 tumors, based on the following parameters: (1) tumor growth rate; (2) probability of stage progression as a function of size; (3) probability of clinical diagnosis as a function of size (Figure 1, Method 3). Progression to stage III+ was assumed to be stochastic and size-dependent; each of the 1,000 bootstrap Kaplan-Meier analyses (above) was used to define risk of progression as a function of size for one of the 1,000 model tumors. Clinical diagnosis was assumed to be stochastic and size-dependent. For high-risk women, the probability of clinical diagnosis as a function of size was based on the size distribution of serous tumors detected in a large trial of screening for early detection [35]. For normal-risk women, we estimated the probability of clinical diagnosis as a function of size by assuming that tumors in this population are clinically diagnosed at diameters log-normally distributed around a mean of 8 cm. The R code used for this analysis is available in Text S2.

### Estimating Sensitivity of Early Detection Testing

We estimated the sensitivity of hypothetical screening scenarios by performing a hypothetical screening test in the 1,000 model tumors described above (Figure 1, Method 4). We evaluated sensitivity requirements as a function of the interval between tests (3, 6, 12, or 24 mo) and the minimum diameter at which a hypothetical test can detect a tumor. We assumed that the test would always fail to detect an occult tumor below the size threshold and would always succeed when the tumor size exceeded the size threshold. Tests were considered positive when a “progressive tumor” (a tumor that would be diagnosed at an advanced stage in the absence of screening) was above the size threshold for detection but still less than stage III at the time of one of the screening tests. Sensitivity in early detection was defined as the number of true positive tests (i.e., tumors detected before progression to stage III or IV) divided by the number of “progressive tumors” (tumors that would be diagnosed at an advanced stage in the absence of screening). The R program used for this analysis is available in Text S2.

### Estimating Survival Benefits of Early Detection Testing

We estimated the 5-y survival benefits of hypothetical screening scenarios by performing of a hypothetical screening test in the 1,000 model tumors described above, and combining the resulting stage distribution with stage-specific survival data (Figure 1, Method 5). We evaluated survival benefits as a function of the interval between tests (3, 6, 12, or 24 mo) and the minimum diameter at which a hypothetical test can detect a tumor. We assumed that the test would always fail to detect an occult tumor below the size threshold and would always succeed when the tumor size exceeded the size threshold. We estimated the 5-y survival benefit of a given screening scenario by comparing mortality in the absence of screening to the mortality estimated according to the distribution of stages of tumors detected with screening given available stage-specific survival data for serous cancers from SEER (1990–2004) [36]. The R program used for this analysis is available in Text S2.

### CIs for Model Outputs: Tumor Growth Rates, Sensitivity, and Reduction in 5-y Mortality

We derived CIs for our estimates of early detection test performance requirements based on 200 iterations of our entire modeling procedure (Figure 1). In each of the 200 iterations, we generated a set of input occult tumor sizes (*n* = 37) by sampling with replacement from the original set of 37 literature-derived values (Figure 1, Data 1). We then repeated the Kaplan-Meier analysis of stage progression as a function of tumor size (Figure 1, Method 1), and Monte Carlo modeling of tumor growth (Figure 1, Method 2) described above, for each set of 37 tumor sizes. Next, for each of the resulting sets of functions, we generated a set of 25 model tumor life histories (Figure 1, Method 3) (i.e., 200 sets of 25 tumors). Each model tumor was assigned an occult period duration and a size at clinical diagnosis by sampling with replacement from the appropriate distribution (see Methods, data sources above).

By applying a hypothetical annual screen to each of these 200 sets of 25 model histories, we obtained 200 estimates of sensitivity (Figure 1, Method 4) and 200 estimates of the improvement in 5-y survival (Figure 1, Method 5), as a function of the threshold tumor size for detection. We obtained CIs directly from the results of the 200 iterations (see Figure S5). In Results, we report the 95% confidence intervals for the tumor size detection threshold required to achieve a specified sensitivity or mortality reduction (the upper limit of this confidence interval is the tumor size threshold at which the specified sensitivity was achieved in only 5 of the 200 sets of model tumor life histories; the lower limit is the size threshold at which the specified sensitivity was achieved in 195 of the 200 sets of model tumor life histories). In addition, we estimated the probability densities for tumor growth rates based on the 200 growth rate estimates derived as described above (Figure S6).

### Sensitivity of the Model to Changes in Key Input Parameters

We investigated the sensitivity of our model and our major conclusions to systematic errors in the five key inputs: (1) distribution of sizes of serous ovarian tumors at clinical presentation in *BRCA1* women; (2) distribution of sizes of serous ovarian tumors at clinical presentation in the general population; (3) duration of the occult period (e.g., due to sampling error or systematic biases in our estimates of incidence and prevalence); (4) Staging of the tumors discovered by PBSO; and (5) Fraction of occult early-stage tumors that if left intact would ultimately be diagnosed as ovarian cancer. In each case, the analyses were performed by repeating the entire modeling procedure as outlined in Figure 1, using altered values for the parameters in question. For the first three of these parameters, we examined the effects of varying the respective values between 50% and 200% of the best available estimates from the data (our primary modeling inputs). For staging, we examined how our conclusions would be altered if we assumed that randomly selected subset comprising 0% to 100% of the occult tumors discovered by PBSO were understaged by one FIGO stage (i.e., stage I tumors were erroneously classified as CIS, stage II tumors were erroneously classified as stage I, etc.). The effects of changes in these variables on the tumor size detection threshold needed to achieve a given level of early detection test sensitivity or mortality benefit are shown in Figures S7 and S8, respectively.

## Results

The foundation of this study is a synthesis of data from published studies describing occult serous ovarian cancers discovered during prophylactic bilateral PBSO in *BRCA1* women. First, we summarized the characteristics of occult serous ovarian tumors on the basis of these studies. Second, we built on these results to model tumor growth, progression, and diagnosis. Lastly, we examined the performance of hypothetical early detection tests based on our tumor models. The data sources and modeling approach are outlined in Figure 1.

### Characteristics of Occult Serous Ovarian Cancers

We based our analysis of occult serous cancers on studies that met the following criteria: (1) the prophylactic procedure included removal and microscopic examination of both ovaries and both Fallopian tubes; (2) tumor histology was reported; (3) patient genotype was reported; (4) genotyping was done prior to cancer diagnosis (for prevalence estimates only). Table 1 summarizes the PBSO studies that met these criteria. By combining results across studies, we obtained estimates for the prevalence, location, size, and stage distribution of occult serous ovarian cancers in *BRCA1* women (Table 2; Figure S2).

**Table 1:** Sources of occult tumor data (PBSO studies).

Study	*BRCA1* Carriers (*n*)	Occult Serous *BRCA1* Cancers (*n*)	Early Occult Serous *BRCA1* Cancers (*n*)	*BRCA1* Cohort Age (Mean)^a^	Included in Prevalence^b^
Finch et al., 2006 [15]	94	6	6	47	Y
Callahan et al., 2007 [12]	60	2	2	43	Y
Olivier et al., 2004 [21]	58	4	2	46	Y
Laki et al., 2007 [16]	56	3	3	48	Y
Powell et al., 2005 [22]	43	5	5	47	Y
Lamb et al., 2006 [17]	40	5	4	47	Y
Carcangiu et al., 2006 [13]	37	6	4	50	Y
Lu et al., 2000 [19]	18	1	1	46	Y
Colgan et al., 2001 [14]	27	1	1	NS	N
Medeiros et al., 2006 [20]	6	1	1	55	N
Agoff et al., 2000 [11]	<29	3	2	NS	N
Total	—	37	31	—	**—**

a Median age was reported when mean age was not available. When mean age was not given for the *BRCA1* subset, the mean age of the entire series is reported.

b See Table S1 for rationale for exclusion/inclusion from prevalence calculations.

N, no; NS, age was not specified; Y, yes.

**Table 2:** Summary of key characteristics of occult tumors in BRCA1 women.

**Characteristics**		
**Prevalence of occult tumors**		
	Cancers/patients, *n*	32/406
	Mean prevalence (95% CI)	7.9% (5.6%–11.0%)
**Tumor size (early stage)**		
	Tumors, *n*	31
	Median diameter (95% CI)	3.0 mm (2.5–4.4 mm)
**Tumor location, % (** ***n*** **)**		
	FT, % (*n*)	59% (22)
	Ovary, % (*n*)	24% (9)
	FT and ovary, % (*n*)	11% (4)
	Peritoneal, % (*n*)	5% (2)
	Total, *n*	37
**Tumor stage**		
	CIS, % (*n*)	24% (9)
	Stage I, % (*n*)	43% (16)
	Stage II, % (*n*)	16% (6)
	Stage III, % (*n*)	14% (5)
	Stage IV, % (*n*)	3% (1)
	Total, *n*	37

Abbreviation: FT, Fallopian tube.

### The Window of Opportunity for Early Detection

The duration of the “window of opportunity” for early detection—the time during which detection of a tumor can be acted upon to save lives—is a critical parameter in designing an effective early detection strategy. We estimated the duration of each stage in the preclinical progression of serous ovarian tumors by dividing the prevalence of occult cancers at each stage by the incidence of clinically diagnosed serous ovarian cancer (irrespective of stage) in a population matched for age (45.7 y for prevalence; 46.9 y for incidence) and genetic risk (*BRCA1* mutation carriers) [27]. In the eight studies that met our inclusion criteria for calculating the prevalence of occult serous cancers in women with *BRCA1* mutations (Table 1), 32 occult serous cancers were found in 406 PBSOs for an estimated prevalence of 7.9% (95% CI 5.6–10.9%). In the two studies that met our inclusion criteria for calculating incidence, there were 36 serous ovarian cancers in 2,345 woman-years, for an estimated overall incidence of 1.5%/y (95% CI 1.1–2.1/y) (Figure S2; Table S2) [8],[32].

The prevalence/incidence calculation yielded an estimate of 5.1 y (95% CI 3.2–8.1 y) for the median duration of the entire occult period (clinically occult but potentially detectable by meticulous histopathology). Similarly, we estimated the window of opportunity for detection of early stage occult cancers (CIS, stage I, II) to be 4.3 y (95% CI 2.6–6.9 y), and estimated that most serous cancers had progressed to an advanced stage (stage III or IV) a median of 0.8 y (95% CI 0.4–1.9 y) prior to detection (see Figure S2).

### Modeling Tumor Growth, Progression, and Diagnosis

#### Estimating growth rates of occult serous ovarian cancers

We estimated the growth rates of early-stage (CIS, stage I and stage II) and late-stage (stage III and IV) tumors using a Monte Carlo method to fit an exponential growth model to observed data (see Methods). The results suggest that early-stage tumors double in volume, on average, approximately every 4 mo (10-fold per year), and that late-stage tumors double in volume approximately every 2.5 mo (30-fold per year) (Figures S4 and S6).

#### Modeling tumor progression as a function of size

**Figure 2:**
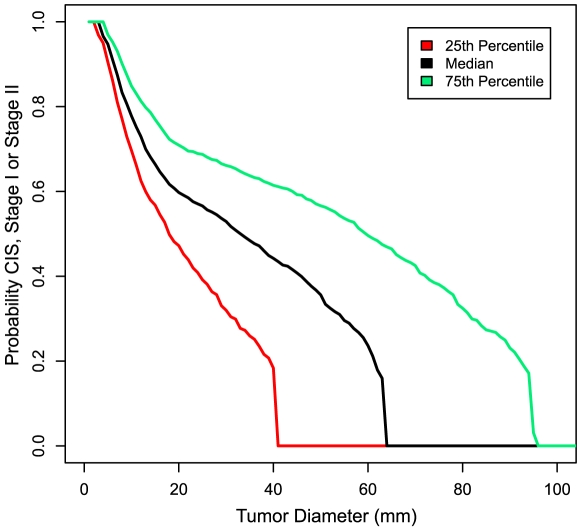
Tumor progression to late stage as a function of tumor diameter. Kaplan-Meier analysis was used to model tumor progression to late stage (III or IV) as a function of tumor diameter (see Methods). Observations were censored at the size of the tumor when discovered by PBSO. CIs are shown as indicated by the legend.

We used a Kaplan-Meier analysis to model tumor progression to late stage (stage III or IV) as a function of tumor size (see Methods). This approach was analogous to a conventional Kaplan-Meier analysis of progression as a function of time after diagnosis, but replacing “time after diagnosis” with “tumor size,” censoring observations at the size of the tumor at diagnosis (i.e., when discovered by PBSO). This analysis indicated that more than 50% of serous tumors advanced to stage III/IV by the time they reached 3 cm in diameter (Figures 2 and S3).

### Estimating Early Detection Test Performance Requirements

We used a Monte Carlo method to model the life trajectories of 1,000 serous cancers, on the basis of the growth parameters and estimates of progression risk as a function of tumor size described above, and estimates of the distribution of tumor sizes at clinical presentation (Figure S1; Table S3) [35]. We then evaluated the performance of hypothetical early detection strategies by applying them to these 1,000 model tumors.

### Estimating Early Detection Test Sensitivity Requirements

We assessed the performance requirements for an early detection test by analyzing the sensitivity of hypothetical screening tests as a function of the limit of detection of the test (tumor diameter) and the interval between screens. We defined successful early detection as detection of a tumor at stage II or earlier that would otherwise be diagnosed at stage III or IV. This analysis was based on our models for growth stage progression and tumor size at clinical presentation as described above.

**Figure 3:**
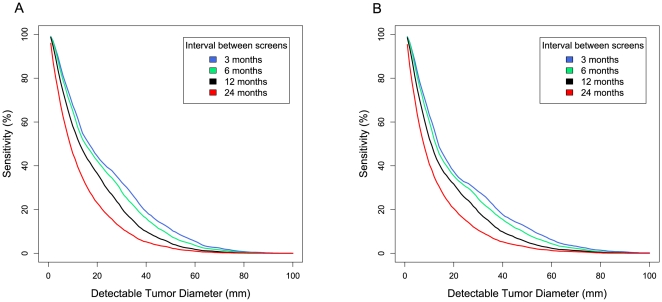
Early detection sensitivity as a function of tumor size detection threshold. The predicted sensitivity of a hypothetical early detection test is shown as a function of the tumor size detection threshold and frequency of a hypothetical screening test (see Methods). Results are shown for two populations of women: (A) Normal-risk women receiving normal care and monitoring; (B) High-risk women subjected to careful monitoring (e.g. *BRCA1* mutation carriers).

Our results indicated that in order to reach an overall sensitivity of 50% in a normal-risk population, an annual screen would need to reliably detect tumors of 1.3 cm or less in diameter. For a sensitivity of 80%, the test would need to detect tumors of 0.4 cm or less in diameter (Figure 3A). In a high-risk population subject to careful conventional monitoring (i.e., similar to that in the screening study described in [35]), an annual screen would need to detect tumors of 1.1 cm and 0.4 cm to reach 50% or 80% sensitivity, respectively (Figure 3B).

### Estimating the Survival Benefit of Early Detection

**Figure 4:**
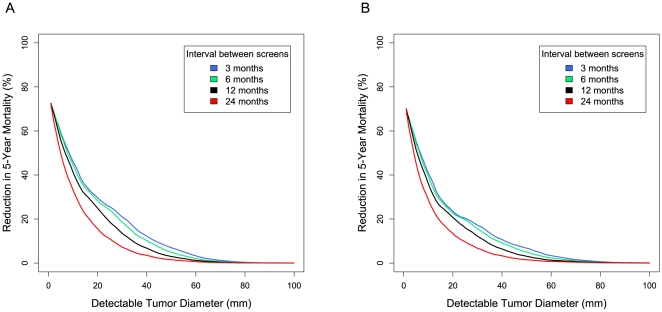
Reduction in 5-y mortality as a function of tumor size detection threshold. The predicted reduction in 5-y mortality from serous ovarian cancer is shown as a function of the tumor size detection threshold and frequency of a hypothetical screening test (see Methods). Results are shown for two populations of women: (A) Normal-risk women receiving normal care and monitoring; (B) High-risk women subjected to careful monitoring (e.g., *BRCA1* mutation carriers).

We estimated the survival benefits of a hypothetical early detection test using stage-specific survival data for serous ovarian cancers [36], and a modeling strategy similar to that described above for analyzing test sensitivity. Again, the key variables we examined were size threshold for tumor detection and frequency of a hypothetical early-detection screening test. The results of this analysis suggest that a 50% reduction in 5-y mortality from serous ovarian cancer in either a normal-risk (Figure 4A) or a high-risk population (Figure 4B) could be achieved with an annual screen capable of detecting a 0.4-cm tumor.

### Potential Effects of Sampling Error and Systematic Biases in the Data

We critically examined the sensitivity of our conclusions to biases and uncertainties in the information on which they are founded. We addressed both the potential effects of systematic errors in empirically derived input parameters of our model, and the confidence ranges of our major conclusions given the limited number of tumors on which our analysis was based.

To determine CIs for our estimates of sensitivity and mortality reduction, we repeated our modeling procedure (see Figure 1), using bootstrap samples of the literature-derived occult tumor (size, stage) values, and durations of the occult interval sampled from its probability distribution (see Methods). Based on these analyses, the 95% CI for the detection threshold required to achieve 80% sensitivity in early detection with an annual screen was 0.1–1.7 cm; for 50% sensitivity, the 95% CI for the required detection threshold was 0.4–3.2 cm (in normal-risk women). The 95% CI for the detection threshold required to achieve a 50% reduction in 5-y mortality with an annual screen was 0.1–2.3 cm for normal-risk women and 0.1–2.1 cm for high-risk women (see Figure S5).

Perhaps the most likely systematic error in the data on which we based our model was understaging of the occult tumors discovered by PBSO. Although we assumed in our model that the stages of all the occult tumors were correctly reported (i.e., 0% understaging), it is likely that a significant fraction of these cancers were understaged, as an overwhelming majority (>90%) were only discovered postoperatively by microscopic examination, indicating that surgical staging was incomplete. Our sensitivity analysis shows that, if understaging of the occult tumors was a significant problem, our conclusions regarding the performance requirements for early detection are likely to be overly optimistic (Table 3; Figures S7C and S8C).

**Table 3:** Sensitivity analyses: effects of systematic errors in model inputs.

	Results with Default Values^a^	Percent Understaging		Size at Clinical dx (*BRCA1*)		Size at Clinical dx (Normal-Risk)		Duration of Occult Period		Percent Progressive Tumors
Input value^b^	—	20%	50%	0.5×	2×	4 cm	16 cm	2.5 y	10.2 y	50%
50% ED sensitivity (cm)^c^	1.1	0.7	0.3	1.2	1.2	0.7	1.7	0.9	1.5	0.9
50% mortality reduction (cm)^d^	0.5	0.2	<0.1	0.5	0.5	0.3	0.6	0.3	0.6	0.3

a Default values were as follows: percent understaging, 0%; size at clinical diagnosis (dx) (*BRCA1*) (see Table S3, reference [35]); size at clinical diagnosis (normal risk), 8 cm diameter; duration of occult period, 5.1 y (see Results); percent progressive tumors, 100%.

b Values shown in first row were used as inputs into the model shown in Figure 1. All other variables were fixed at default values (see footnote a).

c Values indicate the size of tumor (diameter) that an early detection test must detect in order to achieve 50% early detection sensitivity given the modified model input specified in the relevant column header.

d Values shown indicate the size of tumor (diameter) that an early detection test must detect in order to achieve a 50% reduction in 5-y mortality given the modified model input specified in the relevant column header.

A second potential source of systematic bias is our estimate of the distribution of tumor diameters at diagnosis, since these results were based on limited available data (see Methods, data sources). We found that increasing or decreasing our estimates of tumor sizes at clinical presentation in *BRCA1* mutation carriers or in normal-risk women over a 2-fold range had a negligible effect on our conclusions (Table 3; Figures S7A, S7B, S8A, and S8B).

Third, we investigated the effects of errors in our estimate of the duration of the occult period, which could potentially arise because of systematic biases in the data on which we based our estimates of incidence or prevalence (see Methods, data sources). Doubling or halving the estimated duration of the occult period would only slightly alter estimates of the detection threshold required to achieve a given early detection test sensitivity or mortality reduction (Table 3; Figures S7D and S8D).

Lastly, we considered the possibility that a fraction of occult cancers, if left untreated, would never progress to stage III or IV. Our default assumption was that all of the occult cancers would progress and grow to become clinically significant, yet there is evidence that a significant fraction of occult cancers at other sites (e.g., prostate, kidney, and breast [37]) never progress to a clinically significant stage. If instead only 50% of tumors were destined to be “progressive,” a screening test would need to detect tumors at a slightly smaller size to achieve an equivalent early detection sensitivity or mortality reduction (Table 3).

## Discussion

Our challenge was to understand what serous ovarian cancers look like before signs or symptoms lead to their clinical detection. PBSOs, in women with no evidence of serous cancer, provide the only immediately available window on these occult cancers. By aggregating and analyzing the results of all available prospective PBSO studies, we estimated the size and stage distribution of occult serous ovarian cancers, the duration of the “window of opportunity” for early detection, and the location of occult serous cancers (see Figure 1).

With respect to tumor stage, the results were encouraging for early detection; most occult cancers in *BRCA1* carriers were still at an early stage, either CIS, stage I, or stage II. It is important to note, however, that an overwhelming majority (>90%) of these cancers were only discovered postoperatively by microscopic examination, indicating that surgical staging was incomplete; it is therefore likely that some of these cancers were understaged. Indeed, several studies have reported development of primary peritoneal serous cancers within a few years of a PBSO in which no malignancy was found, suggesting that some cancers are missed or understaged in these studies [7],[8],[10],[13],[14],[22].

Our analysis of the duration of time that tumors spend at each stage while still occult was also encouraging with regard to the feasibility of early detection. By comparing the prevalence of occult tumors at each stage to the incidence of serous ovarian cancer in a matched population of women (*BRCA1* carriers, similar age), we estimated that serous tumors spend approximately 4.3 y as histopathologically detectable but clinically occult early stage tumors. This 4.3-y window represents the period of time during which we could presumably save lives through early detection.

We also estimated that, on average, serous ovarian cancers have already progressed to a late stage nearly 1 y prior to their discovery. This conclusion is consistent with the results of several studies of *BRCA1* carriers, which found that most serous cancers present at an advanced stage [32],[38],[39], and with screening studies, which also find that most of these cancers are already advanced when discovered, whether by conventional screening or by symptomatic presentation [23],[40]–[49]. Presumably, the median interval between progression to advanced stage and clinical diagnosis, and the overall duration of the occult period, would be correspondingly longer in women who are monitored less vigilantly than the high-risk women on which we based this analysis.

With respect to tumor size, our results were sobering: we estimated that occult, early-stage (CIS, stage I, or stage II) serous ovarian cancers (in *BRCA1* women) have a median diameter of less than 0.3 cm and spend, on average, more than 90% of the duration of the window of opportunity for early detection at a diameter of less than 0.9 cm (Figure S9). Indeed, many occult cancers had already progressed to stage III or IV when discovered at PBSO, and the majority of these advanced cancers were 1 cm in diameter or smaller. Remarkably, more than 90% of the occult cancers were missed both during surgery and on gross examination and discovered only upon microscopic examination (Table S1).

It is also worth noting that our analysis adds to a growing body of evidence that “ovarian” cancers occur frequently in the Fallopian tubes—we found that occult serous “ovarian” cancers in *BRCA1* mutation carriers are approximately twice as likely to be found in the Fallopian tubes as in the ovaries [14],[15],[18],[20],[22].

We further used the PBSO data, together with data on tumor sizes at clinical diagnosis, to build statistical models for the growth, progression, and diagnosis of serous ovarian cancers. The results of our growth rate estimation highlighted the explosive growth of advanced stage serous ovarian cancers. We estimated that late stage occult ovarian tumors double in volume every 2.5 mo, a conclusion that is reinforced by a previous study that found that most advanced high-grade serous tumors were undetectable by transvaginal ultrasonography several (2–12) mo prior to diagnosis, despite their relatively large size (mean 5.8 cm) at diagnosis [50]. It's interesting to note that if we assume that early-stage serous ovarian cancers grow exponentially at a constant rate beginning with the first cancerous progenitor cell, and that the cancer cells are roughly 15 µm in diameter, extrapolation from this growth model suggests that on average these cancers “originate” about 8 y prior to progression to stage III, and 9 y prior to clinical diagnosis.

We estimated the performance of hypothetical screening tests as a function of their tumor-size threshold for detection and of screening frequency using our models for tumor growth, progression, and diagnosis. Our most important conclusion with respect to early detection was that an annual screening test of normal-risk women would need to detect tumors less than 1.3 cm in diameter in order to achieve 50% sensitivity, or less than 0.4 cm in diameter to reach 80% sensitivity in detecting these cancers before they progress to stage III+ (Figure 3).

Combining the modeling results with stage-specific survival statistics suggests that to achieve a 50% reduction in 5-y mortality from serous ovarian cancer in a normal-risk population, an annual screen would need to detect tumors 0.5 cm in diameter or smaller (Figure 4). Our estimates of potential survival benefits of early detection assumed that the stage-specific survival rates for screen-detected tumors would be the same as those for tumors diagnosed on the basis of signs and symptoms; however, it is quite possible that detecting even advanced tumors at a smaller size might yield additional survival gains. Our analysis of potential mortality reductions from screening ignored the effects of lead-time bias on stage-specific 5-y survival rates, and did not take into account the potential hazards of overdiagnosis. Had we considered either of these factors our estimates of the survival gains for a given early-detection test performance would almost certainly have been lower.

Sensitivity analyses (Table 3; Figures S7, S8) suggest that our major conclusions regarding the performance requirements for a successful early detection strategy are remarkably robust; even improbably large systematic biases or sampling errors in the critical data-driven parameters of the model, (size distribution of tumors at diagnosis, extent of understaging, duration of the occult period), lead to only slight changes in the results, which do not alter the important conclusions.

We based our analyses on women carrying a mutation in *BRCA1*, as they comprise the largest reasonably homogenous defined subset of women who undergo PBSO. The extent to which our findings apply to other ovarian cancers is uncertain. Clearly, it would be a mistake to assume that they apply to ovarian cancers of histological types other than serous; these are distinctly different diseases, clinically and molecularly [3],[4]. Whether our findings in *BRCA1* carriers can be generalized to serous ovarian cancers in general, including sporadic or non-*BRCA1* familial cases, is still an important question. We were able to assess the latter directly, using data from *BRCA2* and other unspecified familial cancers, and found no significant difference in our estimates of prevalence, incidence, duration, size distributions, or locations of occult cancers between these high-risk populations (Table S4). Furthermore, on the basis of current literature, the cancers in women with *BRCA1* mutations appear to be a reasonable model for sporadic serous ovarian cancers. Several studies have compared hereditary and sporadic ovarian cancers and found few significant differences in either clinical or molecular characteristics. The most consistently reported difference is in the distribution of tumor histologies; *BRCA1* mutation carriers tend almost exclusively to develop serous ovarian cancer, whereas a considerable fraction of sporadic ovarian cancers are of endometrioid, mucinous, or clear-cell histology [38],[39],[51],[52]. A few studies have reported small differences in survival and in grade between sporadic and hereditary serous ovarian cancers [38],[53]. Unfortunately, most studies comparing sporadic to hereditary cancers failed to stratify results by histological type, making it difficult to compare serous cases in *BRCA1* mutation carriers to their sporadic counterparts. Although we cannot take for granted that serous ovarian cancers in general can be represented by the natural history model described here, we are not aware of any data regarding sporadic serous cancers that is inconsistent with this model. Moreover, irrespective of their value as models for sporadic serous cancers, understanding the early natural history of serous cancers in *BRCA1* women is important. These women account for up to 10% of all serous ovarian cancers and, as a readily identified high-risk population, are likely to be the first group in which any potential early detection strategy will be evaluated.

For many cancers, early detection is widely believed to be the most promising strategy to save lives [6]. The parameters that we estimated here based on a review of PBSO studies, and the resulting model for the natural history of ovarian cancer, have important implications for rational design of an early detection strategy. The relatively long window of opportunity for detection prior to progression to stage III or IV suggests that an annual screen could be a viable screening strategy. Indeed, a universal annual screen capable of detecting serous cancers 0.5 cm in diameter might reduce 5-y mortality from this disease by 50% (Figure 4). However, our analysis suggests that in order for an annual screen of normal-risk women to achieve a moderate 50% sensitivity in early detection of cancers that would otherwise be diagnosed at an advanced stage, the assay would need to reliably detect tumors of 1.3 cm in diameter (Figure 3).

The need to detect very small tumors has important consequences for both the type of biomolecule and the type of assay technology that are likely to be effective in an early detection strategy. For a blood-based biomarker to be useful for early detection, there must be a significant difference between the maximum biomarker levels observed in the blood of almost all cancer-free women and the levels characteristically observed in women with early, occult cancers. The incremental change in biomarker levels in the blood as a consequence of a 0.5-cm tumor is likely to be extremely small, implying that the baseline levels must be consistently lower still in an overwhelming majority of ovarian cancer-free women in order for the difference to be detectable and robust. A recently published mathematical model relating secreted blood biomarker levels to tumor sizes illustrates that tumors in the millimeter diameter range can only be detected under ideal conditions of extremely high rates of biomarker secretion by tumor-associated cells and essentially zero background from healthy cells; under more realistic conditions, using known parameters of CA125 and PSA, detection limits are in the several to many centimeter diameter range [54]. An addition implication of the need to detect tiny tumors is that biomarker discovery and validation will both require extremely sensitive biomarker assays. This requirement is especially challenging in the discovery phase, particularly for proteomic markers, as mass spectrometry-based proteomics approaches are only recently dipping into the nanogram/milliliter range for detection and quantification—well above the range of serum concentrations expected for proteins produced exclusively by subcentimeter-sized tumors.

Most published studies have evaluated ovarian cancer biomarkers on the basis of their ability to detect ovarian cancer in women with clinically apparent tumors [55]–[57]. An important implication of our analysis is that these clinically apparent tumors are not good models for the tumors that an effective early detection test would need to detect. Indeed, our analyses show that early-stage serous ovarian cancers are rarely more than a few millimeters in diameter. In contrast, the infrequent serous cancers that present clinically while still stage I or II are typically 8 cm or more in diameter (P. Shaw and B. Rosen, unpublished data). Thus, by the time they become clinically apparent, most serous ovarian cancers are more than 200 times larger than the presymptomatic tumors a successful early detection strategy must detect. We must therefore be extremely cautious in extrapolating from the performance of serum biomarkers in detecting clinically apparent tumors to utility in early detection. Unfortunately, the magnitude of this leap is often ignored; many reports of candidate blood biomarkers for ovarian cancer assume that the detection of symptomatic stage I and II tumors will translate to an effective early detection test. Some have even used this fallacious reasoning to promote useless commercial tests [56],[58]. None of the biomarkers or biomarker panels reported to date has come close to demonstrating the performance that, according to our analysis, would be required for useful early detection. Indeed, the few large prospective trials of screening for ovarian cancer have yielded disappointing results [35],[45],[59],[60]

It is likely that some combination of new biomarkers and new approaches will be needed to meet the challenge of early detection. Novel biomarkers that are truly unique to cancers (e.g., RNA or protein products of oncogenic gene fusions)—if they exist—are one attractive possibility. Detection of tumor biomarkers in alternative biospecimens like proximal fluids (e.g., for ovarian cancer, vaginal, or uterine lavage), rather than in blood, may be a useful strategy for boosting signal to noise by both reducing background from nonmalignant tissues and avoiding the problem of biomarker dilution inherent in blood-based assays. Positron emission tomography (PET) or ultrasound imaging of specific molecular targets is another potentially promising approach to early detection of ovarian cancers currently under investigation [61],[62]. Identification of adequately specific molecular markers and development of assays or imaging probes that provide the necessary sensitivity and signal to background ratio is still a great challenge.

Given the critical importance of the parameters that we estimated here for rational design of an early detection strategy, we believe that it would be extremely valuable to perform similar investigations into the early natural history of ovarian cancer in normal-risk women, and of other cancers. In theory, it should be possible to characterize occult “ovarian” cancers in the normal-risk population by incorporating meticulous microscopic examination of the ovaries and Fallopian tubes into the pathological examination of the resected tissues from routine hysterectomies, as over 600,000 such operations are performed annually in the United States alone (approximately half of which include salpingo-oophorectomy [63]). For other cancers that develop in organs that are rarely or never removed from healthy individuals, large-scale autopsy studies including meticulous microscopic examination of target organs could potentially provide the necessary data. Such a strategy would be extremely challenging, due not only to the significant cost, but also to the relative rarity of autopsies today, the lack of detailed microscopic examination of apparently normal tissues, and the lack of a coordinated framework for systematically collecting data from autopsies. Nevertheless, we believe that understanding the early natural history of cancer is of such critical importance that the cost and effort would be justified.

## Supporting Information

Figure S1 Size distribution models for serous ovarian cancers at clinical diagnosis. The distributions of sizes (diameters) of tumors at clinical diagnosis are shown for both a high-risk population (i.e., *BRCA1* women); and a normal-risk population. For the high-risk population, tumor diameters were modeled on the basis of sizes reported in a large study of screening for ovarian cancer [35]. The diameters reported in that study were smoothed with a log-normal kernel; for tumors diagnosed in the intervals between screens (whose sizes were not reported in the screening study), the sizes at diagnosis were assumed to be log-normally distributed around a mean of 8 cm. For the normal-risk population, tumor diameters were assumed to be log-normally distributed with a mean of 8 cm.
(0.66 MB EPS)

Figure S2 Probability distributions for true incidence, prevalence, and occult period duration of serous ovarian cancer in *BRCA1* women given the observed data (see Methods). (A) Prevalence; (B) Incidence; (C) Duration of occult period; (D) Duration of early-stage occult period.
(0.97 MB EPS)

Figure S3 Model tumor progression as a function of tumor size. Results of a Kaplan-Meier analysis of 1,000 bootstrap samples of the tumor (size, stage) data for occult serous cancers discovered by PBSO, to estimate the risk of tumor progression (see Methods). Results are shown for progression to stage II+ and for progression to stage III+, with and without normal kernel smoothing of the input tumor size data. (A) Progression to stage II+; with smoothing; (B) Progression to stage II+; no smoothing; (C) Progression to stage III+; with smoothing; (D) Progression to stage III+; no smoothing.
(1.57 MB EPS)

Figure S4 Observed versus model-predicted sizes of occult serous ovarian cancers. (A) Early-stage (CIS, stage I and II) occult tumors and (B) advanced-stage (stage III and IV) occult tumors. Observed sizes were obtained directly from PBSO studies (Table S1). Predicted sizes were obtained from the Monte Carlo simulation of tumor life histories (Figure 1, Method 3) using growth parameters and size-dependent progression models derived using 200 bootstrap samples of the occult tumor (size, stage) data.
(3.81 MB TIF)

Figure S5 CIs for early detection test performance versus tumor detection threshold. Bootstrap CIs are shown for our estimates of early detection test performance requirements as indicated by the legend (see Methods). All results shown here are for an annual screening test. (A) and (B) Early detection test sensitivity versus detection threshold. (A) Normal-risk women; (B) High-risk women (i.e., *BRCA1* women); (C) and (D) Reduction in 5-y mortality versus detection threshold. (C) Normal-risk women; (D) High-risk women (i.e., *BRCA1* women).
(1.07 MB EPS)

Figure S6 Model tumor characteristics. (A, B) Bootstrap probability distributions for tumor growth rates in *BRCA1* women (see Methods): (A) Stage CIS, I, II tumors; (B) Stage III, IV tumors. (C, D) Monte-Carlo models for tumor life histories (see Methods). Fifty model tumor trajectories are shown in each of two populations: (C) High-risk women; (D) Normal-risk women.
(1.51 MB EPS)

Figure S7 Effects of systematic errors in source data on estimates of test sensitivity versus detection threshold. For each of four model input variables, we varied the input over several fold and plotted the resulting estimates of early-detection sensitivity as a function of tumor size detection threshold (see Methods). (A–C) Results assuming annual screening tests. (D) Tumor size detection thresholds needed for 50% sensitivity. (A) Effect of tumor size at clinical diagnosis in normal-risk patients. Our primary analyses assumed a mean tumor diameter at diagnosis of 8 cm. Results are shown for this size as well as the other tumor sizes indicated in the legend. (B) Effect of tumor size at clinical diagnosis in high-risk patients. Our primary analyses assumed that serous ovarian cancers in normal-risk women present at sizes corresponding to the distribution reported in [35]. Results are shown for this size (1×) as well as for the other size distributions indicated in the legend (e.g., 2× = all sizes multiplied by 2 relative to the 1× size distribution). (C) Effect of understaging of tumors found by PBSO. Our primary analyses assumed 0% understaging. Results are shown for no understaging (0%) through 100%, where the indicated percentage of the occult tumors used for our analysis were adjusted to be one FIGO stage more advanced than reported. (D) Effect of variation in duration of occult period. The duration estimate used in our primary analyses is indicated by 1×. “Fold error in duration estimate” is the ratio of the estimated duration to the true duration of the occult period.
(1.32 MB EPS)

Figure S8 Effects of systematic errors in source data on estimates of mortality reduction versus detection threshold. For each of four model input variables, we varied the input over several fold and plotted the resulting estimates of reduction in 5-y mortality as a function of tumor size detection threshold (see Methods). (A–C) Results assuming annual screening tests. (D) Tumor size detection thresholds needed for 50% reduction in 5-y mortality. (A) Effect of tumor size at clinical diagnosis in normal-risk patients. Our primary analyses assumed a mean tumor diameter at diagnosis of 8 cm. Results are shown for this size as well as the other tumor sizes indicated in the legend. (B) Effect of tumor size at clinical diagnosis in high-risk patients. Our primary analyses assumed that serous ovarian cancers in normal-risk women present at sizes corresponding to the distribution reported in [35]. Results are shown for this size (1×) as well as for the other sizes indicated by the fold changes in the legend (e.g., 2× = all sizes multiplied by 2× relative to the 1× size distribution). (C) Effect of understaging of tumors found by PBSO. Our primary analyses assumed 0% understaging. Results are shown for no understaging (0%) through 100%, where the indicated percentage of the occult tumors used for our analysis were adjusted to be one FIGO stage more advanced than reported. (D) Effect of variation in duration of occult period on threshold for 50% mortality reduction. The duration estimate used in our primary analyses is indicated by 1×. “Fold error in duration estimate” is the ratio of the estimated duration to the true duration of the occult period.
(1.31 MB EPS)

Figure S9 Cumulative distribution of sizes of occult early-stage tumors discovered by PBSO. If we assume that these early-stage occult tumors were discovered by PBSO at times uniformly sampled from the window of opportunity for early detection, the cumulative percentage of tumors below a specified size represents the fraction of the window of opportunity during which tumors are less than that size. Thus, this analysis implies that tumors are less than 3 mm in diameter for more than 50% of the window of opportunity for early detection, and less than 9 mm in diameter for more than 90% of this window of opportunity.
(0.63 MB EPS)

Table S1 Detailed findings in PBSO studies on which analyses of occult serous cancers were based.
(0.05 MB XLS)

Table S2 Summary of studies used to estimate incidence of serous ovarian cancer in *BRCA1* women.
(0.04 MB XLS)

Table S3 Summary of tumors used for modeling distribution of sizes at diagnosis in a closely monitored population.
(0.02 MB XLS)

Table S4 Comparison of occult tumor characteristics in *BRCA1* women versus all high-risk women.
(0.02 MB XLS)

Text S1 R program for calculating probability distributions and CIs for prevalence, incidence, and duration of occult period and stages.
(0.02 MB DOC)

Text S2 R program for modeling the preclinical natural history of serous cancer-stage progression as a function of size, growth rates, sensitivity of early-detection screening programs and expected reduction in 5-y mortality as a function of size threshold for tumor detection and screening interval.
(0.02 MB DOC)

Text S3 R program for calculating probability distributions and CIs for prevalence, incidence, and duration of occult period and stages, assuming that 50% of the early tumors discovered by PBSO will never progress to a clinically significant stage (i.e., they do not contribute to incidence or mortality).
(0.08 MB DOC)

Text S4 R program for modeling early-detection sensitivity and changes in 5-y mortality as a function of size threshold for tumor detection and screening interval, assuming that 50% of the early tumors discovered by PBSO will never progress to a clinically significant stage (i.e. they do not contribute to incidence or mortality).
(0.01 MB DOC)

Text S5 R program for calculating probability distributions and CIs for prevalence, incidence, and duration of occult period and stages. This is the same as Text S1, except it runs faster at the expense of a small reduction in the precision of the estimates. Note that the graphs used in Figure S1 were produced using Text S1.
(0.08 MB DOC)

## Abbreviations

CI: confidence interval
CIS: carcinoma in situ
PBSO: prophylactic bilateral salpingo-oophorectomy
