# Supplementary figures and images for: The Preclinical Natural History of Serous Ovarian Cancer: Defining the Target for Early Detection
Source: PLoS Med. 2009 Jul 28;6(7):e1000114. doi: 10.1371/journal.pmed.1000114 (PMC2711307; doi:10.1371/journal.pmed.1000114)

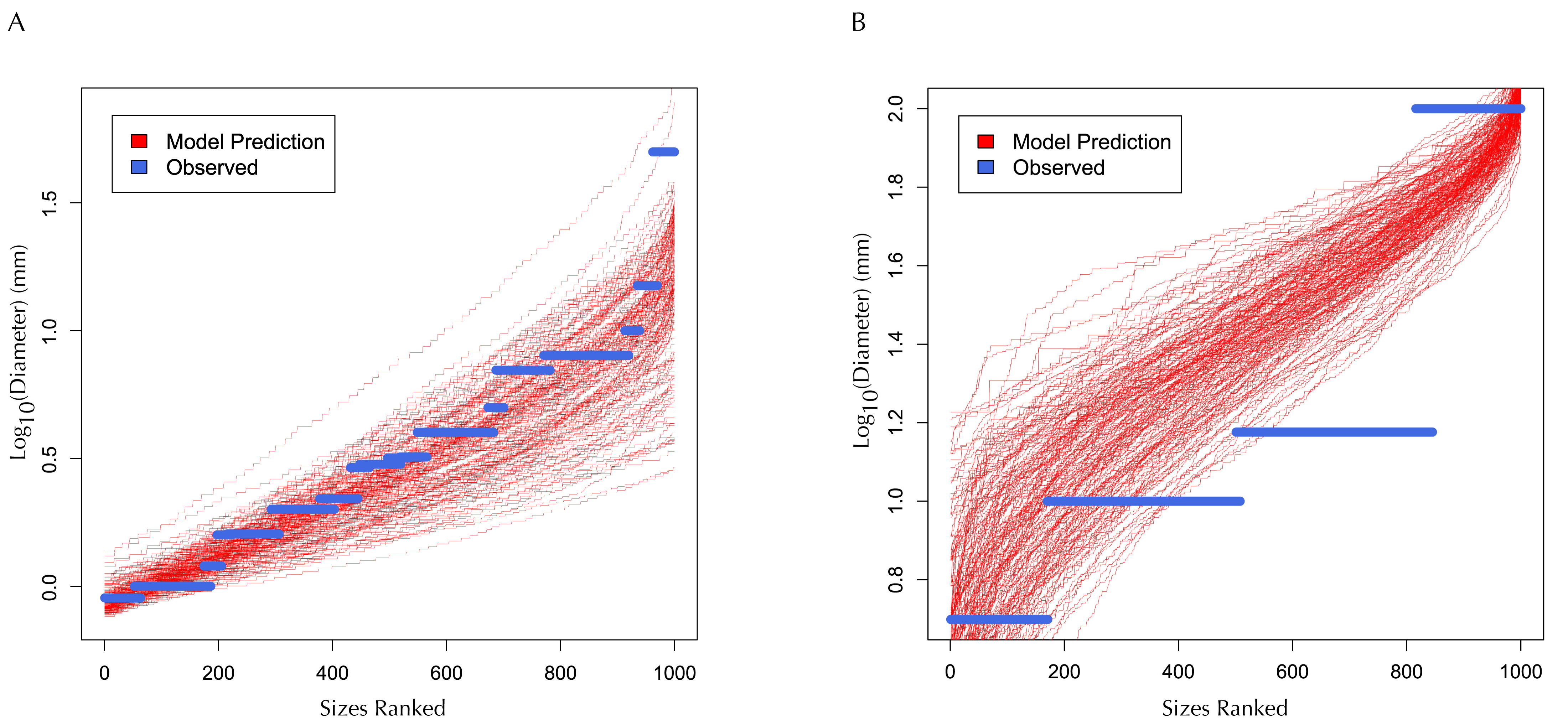

Supplement: Figure S4 — Observed versus model-predicted sizes of occult serous ovarian cancers. (A) Early-stage (CIS, stage I and II) occult tumors and (B) advanced-stage (stage III and IV) occult tumors. Observed sizes were obtained directly from PBSO studies (Table S1). Predicted sizes were obtained from the Monte Carlo simulation of tumor life histories (Figure 1, Method 3) using growth parameters and size-dependent progression models derived using 200 bootstrap samples of the occult tumor (size, stage) data. (3.81 MB TIF) [file pmed.1000114.s004.tif]
